# An Older Woman With Postoperative Breast Metastasis From Ascending Colon Cancer Successfully Treated With Pembrolizumab

**DOI:** 10.7759/cureus.97608

**Published:** 2025-11-23

**Authors:** Masako Mizoguchi, Takaki Furuyama, Ja-Mun Chong, Yusuke Kinugasa, Satoru Iida

**Affiliations:** 1 Department of Surgery, Tokyo Metropolitan Toshima Hospital, Tokyo, JPN; 2 Department of Pathology, Tokyo Metropolitan Toshima Hospital, Tokyo, JPN; 3 Department of Gastrointestinal Surgery, Institute of Science Tokyo, Tokyo, JPN

**Keywords:** colorectal cancer, immune-checkpoint inhibitor, medullary carcinoma, metastatic breast cancer, pembrolizumab

## Abstract

Metastatic breast cancer is sporadic, and colorectal cancer as its primary site is even more uncommon. Here, we report a case of metastatic breast cancer in a patient who had undergone surgery for ascending colon cancer. An 82-year-old woman was diagnosed with a breast tumor that was identified as metastatic by immunohistochemical staining, mainly cytokeratin 20 and cytokeratin 7. Additionally, the patient had developed lymphadenopathy in the abdominal aortic region. Because of microsatellite instability-high (MSI-H) status, pembrolizumab was chosen for treatment, and after 23 cycles, all metastatic lesions were no longer detectable. The diagnosis of metastatic breast cancer is challenging, but immunohistochemical staining facilitated an accurate diagnosis in this case. We also noted that the histological type of this case was medullary carcinoma. Furthermore, as far as we could find out, this is the first reported case of colon cancer metastasis to the breast successfully treated with immune checkpoint inhibitors.

## Introduction

Metastatic breast tumors are uncommon, accounting for approximately 2% of all breast tumors, with primary breast tumors comprising the majority [1]. Among these, metastases originating from colorectal cancer are exceedingly rare, although the exact probability is unclear; only about 50 cases have been reported to date [2]. To date, the standard treatment for metastatic colorectal cancer has consisted of 5-fluorouracil (5-FU)-based chemotherapy regimens such as folinic acid, 5-FU, and oxaliplatin (FOLFOX) and folinic acid (leucovorin), 5-FU, and irinotecan (FOLFIRI), often in combination with molecular targeted drugs [3]. However, in recent years, the immune checkpoint inhibitor pembrolizumab has developed as a new therapeutic option for patients with microsatellite instability-high (MSI-H) colorectal cancer [3]. To our knowledge, there have been no reports of their use in treating breast metastases from colorectal cancer. We present the case of an 82-year-old woman with medullary carcinoma of the colon who developed metastatic disease involving lymph nodes and the breast following surgical treatment for ascending colon cancer and exhibited a favorable response to pembrolizumab.

## Case presentation

**Figure 1:**
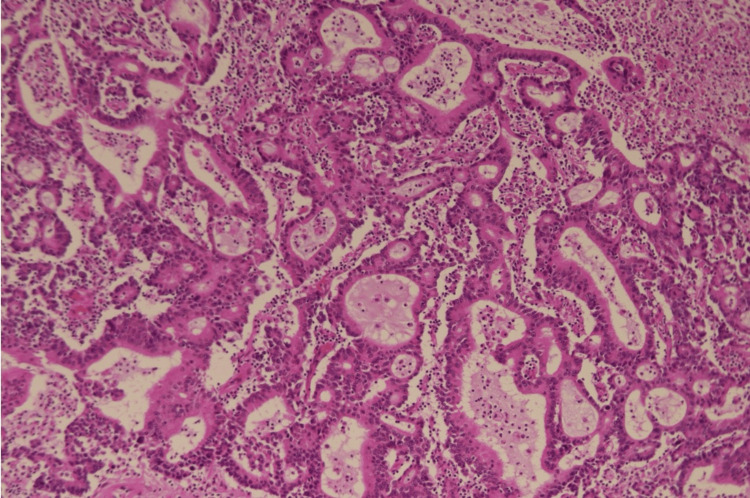
Hematoxylin and eosin (H&E) staining of the colon cancer

**Figure 2:**
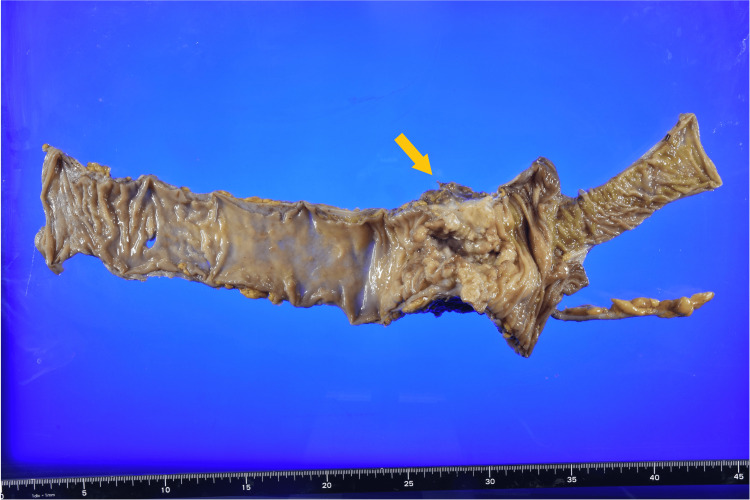
Macroscopic findings of the resected specimen of the colon cancer A bulky tumor is observed in the proximal ascending colon

An 82-year-old woman presented to our hospital with complaints of anemia and a right abdominal mass. The patient’s Eastern Cooperative Oncology Group (ECOG) performance status was 2, and she had hypertension as a comorbidity. Initial computed tomography (CT) imaging revealed a lesion in the ascending colon measuring 7.0 × 6.0 cm. Colonoscopic biopsy confirmed the diagnosis of adenocarcinoma, and staging investigations classified the tumor as T4aN1aM0. Serum CEA level was 8.38 ng/ml, and the CA19-9 level was 430.2 U/ml. The patient underwent laparoscopic right hemicolectomy with D3 lymph node dissection. Histopathological examination of the resected specimen identified medullary carcinoma (Figure 1), with tumor dimensions of 7.0 × 6.0 cm and showing associated ulceration (Figure 2). Immunohistochemical analysis demonstrated the following profile: cytokine (CK)7-partial positive, CK20-partial positive, CDX2-negative, MUC5AC-positive, MUC2-positive, synaptophysin-positive, CD56-positive, RAS wild-type, and BRAF mutation-positive. Metastasis was identified in one regional lymph node.

**Figure 3:**
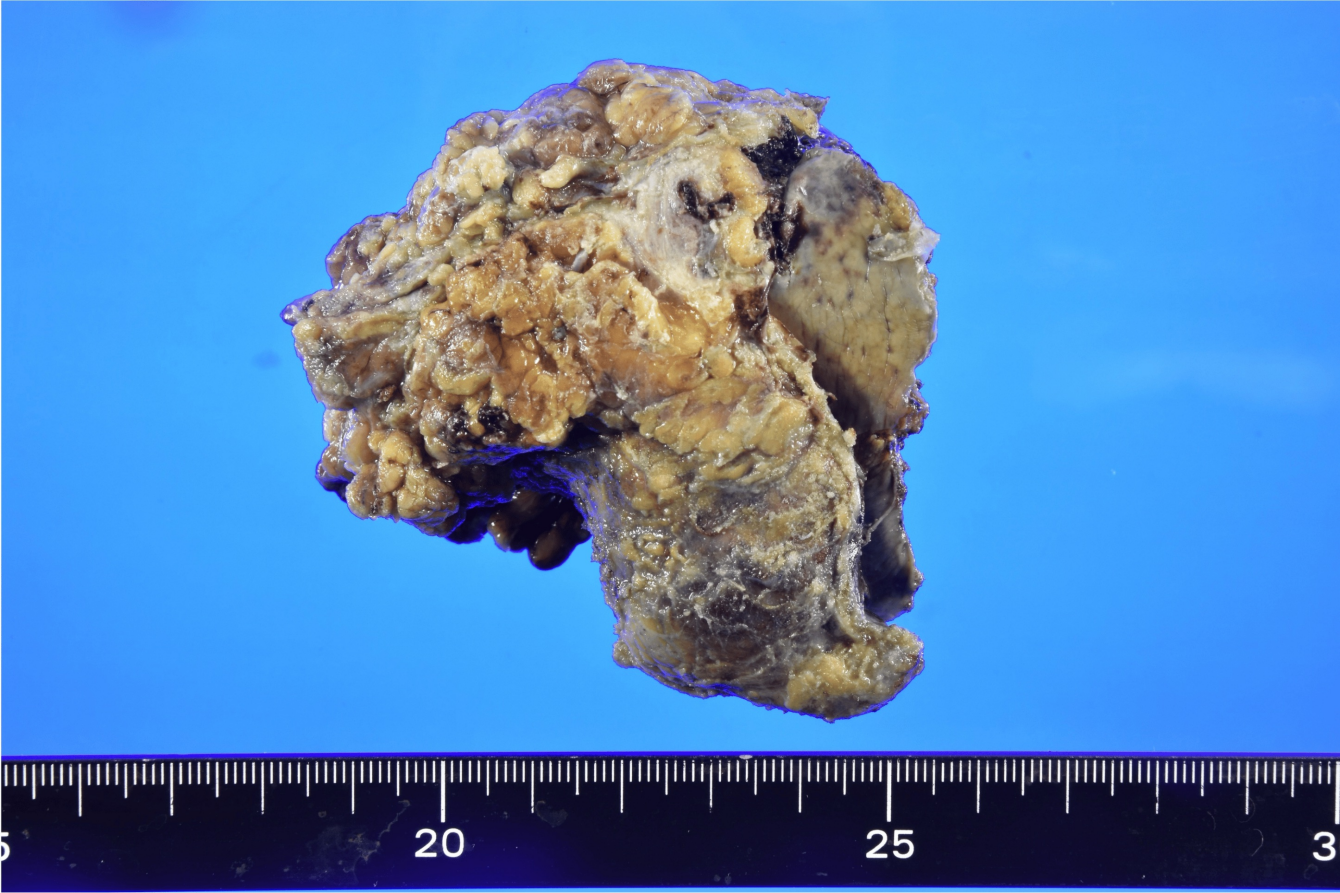
Macroscopic findings of the resected specimen of the liver metastasis

Six months postoperatively, follow-up CT imaging identified a mass with contrast enhancement located at the inferior margin of hepatic segment S6, consistent with hepatic metastasis and peritoneal dissemination. The serum CEA level was 178.3 ng/ml, and CA19-9 was 32.6 U/ml. The patient underwent laparoscopic resection of the hepatic lesion, which measured 5.0 × 4.5 × 4.5 cm (Figure 3).

Immunohistochemical staining of the excised tissue demonstrated features concordant with the primary tumor, including partial CK20 positivity, CK7 negativity, CDX2 negativity, and MUC5AC positivity. Table 1 shows immunohistochemical features of all specimens (Table 1). Additionally, microsatellite instability-high (MSI-H) status was confirmed.

**Table 1:** Immunohistochemical findings of the primary tumor and metastatic lesions All specimens showed strong positive of MUC5AC

Immunohistochemical marker	Primary tumor	Liver metastasis	Breast metastasis
Cytokine (CK) 7	Partially positive	Negative	Negative
Cytoline (CK) 20	Partially positive	Positive	Partially positive
CDX2	Negative	Negative	Negative
MUC5AC	Positive	Positive	Positive

**Figure 4:**
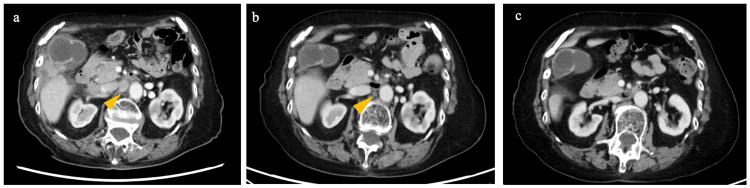
Lymphadenopathy in the abdominal aortic region Nine months after the initial surgery (a), following eight courses (b), and following 27 courses of pembrolizumab treatment (c)

**Figure 5:**
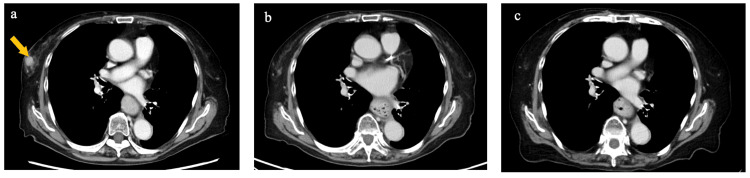
Computed tomography images of breast metastasis At nine months after the initial surgery (a), following eight courses (b), and following 27 courses of pembrolizumab treatment (c). After nine months, the tumor is barely detectable

**Figure 6:**
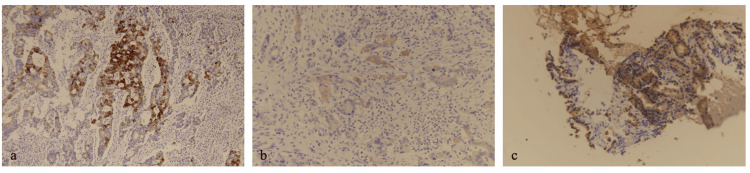
Cytokeratin 20 immunohistochemical staining of the primary tumor (a), liver metastasis (b), and breast metastasis (c)

Nine months following the initial surgery, CT imaging was performed due to the elevation of tumor marker (CEA, 156.84 ng/ml; CA19-9, 73.1 U/ml) and revealed new metastatic disease, including 2.2 cm lymphadenopathy in the abdominal aortic region (Figure 4a) and a 1.5 cm lesion in the right breast (Figure 5a). Mammotome biopsy of the breast lesion revealed histopathological features consistent with colorectal carcinoma metastasis, including partial CK20 positivity, CK7 negativity, CDX2 negativity, and MUC5AC positivity (Figure 6).

Considering the patient's age, ECOG PS, and the MSI-high status of the tumor, pembrolizumab therapy was initiated 10 months postoperatively. At the start of pembrolizumab treatment, serum CEA level was 416.4 ng/ml and CA19-9 level was 265.0 U/ml. After eight courses of treatment, the right breast lesion was no longer detectable on follow-up imaging (Figures 5b,5c). After 23 cycles, para-aortic lymph nodes were no longer visible on CT (Figures 4b, 4c), leading to the determination of a complete response (CR). CEA and CA19-9 had improved to within the normal range at this time (CEA, 4.51 ng/ml; CA19-9, 21.7 U/ml). The patient has now been receiving pembrolizumab treatment for 31 months since the initial surgery, completing a total of 30 courses, with sustained evidence of a CR.

## Discussion

Metastatic breast tumors are an uncommon clinical entity, accounting for approximately 2% of all breast malignancies [1]. The primary sites of origin for these metastases are highly variable and differ across studies. In the largest case series by Williams et al., the skin was identified as the most frequent site of primary malignancy [4]. DeLair et al. reported that the ovaries accounted for 21% of cases [1], while Sun et al. identified the lungs as the most common site, comprising 22.7% of cases [5].

Metastatic breast tumors originating from colorectal cancer are particularly rare, with the incidence reported to be as high as 6% [1]. Hsieh et al. documented 46 cases of mammary metastases from colorectal cancer between 1976 and 2019 [2]. Primary colorectal tumors leading to mammary metastases are more commonly located in the left colon [2]. Furthermore, metastases to other organs are frequently observed in these cases, with 30 of the 46 previously reported cases exhibiting metastases to organs beyond the mammary gland, including 15 cases with multiorgan involvement. 

Management of mammary metastases typically involves a combination of surgical resection and systemic chemotherapy. Chemotherapeutic regimens are predominantly irinotecan- or oxaliplatin-based [2,6]. In many previously reported cases, the effect on improving prognosis was inconsistent; the mean survival time from diagnosis of the mammary metastasis is reported to be 14.9 months [2]. The current case demonstrated a successful response to first-line pembrolizumab, an immune checkpoint inhibitor targeting PD-1, in a patient with MSI-H metastatic colorectal cancer. This represents the first documented use of pembrolizumab for the treatment of mammary metastases. Pembrolizumab has shown superior progression-free survival compared with standard chemotherapy in MSI-H metastatic colorectal cancer [3]. Saberzadeh-Ardestani et al. further highlighted its tolerable adverse event profile in elderly patients and noted prolonged progression-free survival cases of nonliver metastases compared with liver metastases [7]. Immune checkpoint inhibitors, including pembrolizumab, have also been associated with improved overall survival in elderly patients compared with younger cohorts [8]. Notably, in the present case, an 82-year-old patient achieved sustained PR without adverse events during ongoing treatment.

Immunohistochemical staining was conducted to confirm that the three foci represented metastatic lesions. Colorectal cancers are typically characterized by a cytokeratin 7-negative/cytokeratin 20-positive (CK7−/CK20+) profile in 65.8% of cases, whereas primary breast cancers predominantly exhibit a CK7-positive/CK20-negative (CK7+/CK20-) pattern [5,9]. CDX2, a marker expressed in over 90% of colorectal cancers, is absent in mammary glands [5]. In the current case, CDX2 staining was negative in all samples. Conversely, MUC5AC was strongly expressed in all three specimens. 

Medullary carcinoma (MC), a rare histologic subtype of colorectal cancer, accounts for less than 3% of all primary colorectal cancers [10]. It was previously classified under poorly differentiated adenocarcinoma (PDA) before being recognized as a distinct entity in the 2010 WHO Classification of Tumors (3rd edition). Retrospective analyses suggest that some instances initially classified as PDA may represent MC. For instance, Scott et al. reported that 15.3% of cases previously identified as PDA were reclassified as MC [11].

Clinically, MC is more frequently observed in women, older individuals, and tumors located in the right-sided colon [10]. It is strongly associated with BRAF mutations and MSI-H status [12]. In colorectal cancer, BRAF mutation is generally strongly associated with MSI-H, suggesting that MSI-H may be linked to a more favorable prognosis compared with microsatellite stability (MSS) within the BRAF mutation subgroup [13]. Immunohistochemical features distinguishing MC from conventional colorectal carcinoma include prominent positivity for MUC5AC, considered a hallmark of this histological subtype. MUC5AC, one of the gastric mucins, is closely associated with MSI-H colon cancer [14]. Although MC is generally associated with a favorable prognosis, evidence suggests that outcomes may be poorer for patients with Stage III disease or higher compared with other histologic subtypes [15].

Limitations of this case include its single-case nature and lack of reproducibility. Furthermore, some molecular profiles, such as Programmed Death-Ligand 1 (PD-L1) expression and Tumor Mutation Burden (TMB), were not evaluated.

## Conclusions

Mammary metastasis represents an uncommon manifestation of colorectal cancer dissemination. Nonetheless, it should be considered in the differential diagnosis when a newly identified mammary lesion arises in a patient with advanced-stage colorectal cancer. In such cases, treatment should involve close collaboration among a gastrointestinal surgeon, a breast surgeon, a pathologist, and a medical oncologist. Pembrolizumab, with its relatively favorable safety profile, including in elderly patients, may be a viable therapeutic option even for extended treatment durations.
